# Infant attention to rhythmic audiovisual synchrony is modulated by stimulus properties

**DOI:** 10.3389/fpsyg.2024.1393295

**Published:** 2024-07-02

**Authors:** Laura K. Cirelli, Labeeb S. Talukder, Haley E. Kragness

**Affiliations:** ^1^Department of Psychology, University of Toronto Scarborough, Toronto, ON, Canada; ^2^Psychology Department, Bucknell University, Lewisburg, PA, United States

**Keywords:** infant perception, audiovisual synchrony, rhythm, music development, eye-tracking

## Abstract

Musical interactions are a common and multimodal part of an infant’s daily experiences. Infants hear their parents sing while watching their lips move and see their older siblings dance along to music playing over the radio. Here, we explore whether 8- to 12-month-old infants associate musical rhythms they hear with synchronous visual displays by tracking their dynamic visual attention to matched and mismatched displays. Visual attention was measured using eye-tracking while they attended to a screen displaying two videos of a finger tapping at different speeds. These videos were presented side by side while infants listened to an auditory rhythm (high or low pitch) synchronized with one of the two videos. Infants attended more to the low-pitch trials than to the high-pitch trials but did not display a preference for attending to the synchronous hand over the asynchronous hand within trials. Exploratory evidence, however, suggests that tempo, pitch, and rhythmic complexity interactively engage infants’ visual attention to a tapping hand, especially when that hand is aligned with the auditory stimulus. For example, when the rhythm was complex and the auditory stimulus was low in pitch, infants attended to the fast hand more when it aligned with the auditory stream than to misaligned trials. These results suggest that the audiovisual integration in rhythmic non-speech contexts is influenced by stimulus properties.

## 1 Introduction

Music and song are frequently encountered in infants’ everyday soundscapes (Mendoza and Fausey, 2021). While these experiences are sometimes unimodal, such as when infants listen to music from their car seat during a drive, they are often multimodal events. Caregivers gently rock their infants while making eye contact and singing, a melody plays from a rotating mobile above the crib, or a song accompanied by a video plays from a nearby television. A growing body of research suggests that even newborn infants can track an unfolding auditory rhythm (for a review, see Provasi et al., 2014), but many questions remain about how infants integrate auditory rhythms with corresponding visual rhythms and how this integration guides attention over time.

When adults listen to music, synchronous visual displays (e.g., an expressive singer’s face and the performer playing their instrument) have an impact on emotional, perceptual, and esthetic judgments (Schutz and Lipscomb, 2007; Thompson et al., 2008; Platz and Kopiez, 2012; Pan et al., 2019). Adults are also quite capable of detecting audiovisual asynchrony in musical displays, although musical expertise and stimulus features interact to affect task difficulty (Petrini et al., 2009).

Less is known about how and when infants begin to link rhythmic sounds that they hear with synchronous visual displays. The limited research suggests that infants can at least discriminate between synchronous and asynchronous audiovisual rhythmic displays by 6 months of age (Gerson et al., 2015; Hannon et al., 2017). However, beyond discrimination, little is known about how infants deploy attention to competing synchronous or asynchronous audiovisual rhythmic displays when both are present. Hypotheses informed by the auditory scene analysis framework (Bregman, 1994) would predict that infants will deploy visual attention to the object most likely to be creating the auditory stream—for example, a mouth moving in synchrony with the speech stream. This aligns with the intersensory redundancy hypothesis (Lickliter et al., 2017), which stipulates that redundancy in multimodal stimuli effectively recruits attention, facilitating the perception of amodal properties, such as rhythm. Conversely, if detecting audiovisual rhythmic synchrony is easily achieved by infants, they might quickly shift their attention to the asynchronous visual display. This would support information-seeking models of infant attention: for example, the Hunter and Ames (1988) model of infant attention, which predicts that infants will attend to stimuli worthy of continued exploration, as well as the discrepancy hypothesis, which predicts that infants will attend most to events that are moderately complex (Kinney and Kagan, 1976; Kidd et al., 2014). Taken together, these models suggest that infant attention toward rhythmic audiovisual synchrony is likely to be modulated by stimulus properties, such as complexity, and may shift as a scene unfolds over time.

Previous studies reveal substantial variability in infant attention to audiovisual synchrony, potentially stemming from cross-study differences in methodologies, variations in stimulus features (speech vs. non-speech, rhythmic or non-rhythmic), and stimulus complexity (Shaw and Bortfeld, 2015). Existing evidence suggests that very young infants demonstrate an early-emerging preference for synchronous displays. For example, newborns hearing either a vocal or non-vocal sound preferentially look at one of two videos of a vocalizing monkey with a matching temporal structure (Lewkowicz et al., 2010). By around 3 months of age, infants presented with alternating synchronous and asynchronous displays of a face reciting nursery rhymes focused longer on the synchronous displays (Dodd, 1979). Similar synchrony preferences were found in 4-month-old infants watching simultaneously presented synchronous and tempo-shifted displays of two puppets bouncing isochronously and generating impact sounds (Spelke, 1979).

However, studies with older infants and more complex stimuli suggest that audiovisual synchrony does not consistently guide attention across all contexts. For example, when infants listen to an unfolding speech stream alongside two talking faces, infants below 12 months look equally at both displays, whereas those 12 to 14 months look longer at the synchronous display (Lewkowicz et al., 2015). This might suggest that ongoing speech streams are more difficult for infants to associate with competing visual displays compared to the stimuli used in the experiments cited previously. Corroborating this interpretation, the observed preference for synchronous talking faces documented after the first birthday appears to be stimulus-dependent and is eliminated when adult-directed (as opposed to infant-directed) speech or non-native languages are presented (Kubicek et al., 2014). This may be surprising given the early-emerging audiovisual synchrony detection documented even by newborns using vocal stimuli. However, it could be linked to the timing of perceptual narrowing and native-language speech specialization, processes that unfold after 6 months of age (Danielson et al., 2017). Overall, it is unclear whether these results, which seem incongruent with the early emergence of synchrony preference, stem from increased task complexity, developmental changes in cognitive ability (i.e., information-seeking behavior), or properties specific to the stimulus being used.

One stimulus feature of potential importance is pitch. Previous research with adults and infants suggests that listeners focus on high-frequency sounds when identifying the melody of music (Fujioka et al., 2005; Marie and Trainor, 2014; Trainor et al., 2014) and on low-frequency sounds when tracking the rhythm of music (Hove et al., 2014; Lenc et al., 2018, 2023). This suggests that if low-tone rhythms are easier to track (i.e., the low-tone superiority effect), they may also be easier to integrate with synchronous visual displays.

Another potentially important dimension to investigate is how attention is distributed over time. Recent research exploring infant multimodal perception of song, for example, suggests that infants dynamically shift their attention between a singer’s eyes and mouth (Lense et al., 2022). Specifically, infants increase their attention to a singer’s eyes around the musical beat window. These shifting attentional processes, which align with the dynamic attending models of rhythm perception (Large and Jones, 1999), highlight that exploring overall looking patterns collapsed over time may mask indicators of audiovisual integration. Instead, a real-time analysis of infant attention as it unfolds over time, such as with eye-tracking technology, may uncover subtler indications of audiovisual synchrony.

In the present study, we investigated how 8- to 12-month-old infants deploy attention over time while synchronous and asynchronous videos are presented side by side, concurrent with an auditory stimulus. Using eye-tracking, we examined how infants allocated attention to audiovisual synchrony at the trial level. Additionally, we investigated the impact of pitch (high vs. low) on audiovisual integration, given previous observations of a low-tone superiority effect for auditory rhythm processing in infants and adults. Infants were presented with two side-by-side videos depicting a hand tapping with one finger, each playing at distinct rates. Meanwhile, infants listened to either a high- or low-frequency rhythmic pattern synchronized with one of the two videos. We measured infants’ relative looking time to the synchronous and asynchronous videos, as well as the time course of looking as trials unfolded. The auditory scene analysis framework suggests that infants would spend more time looking at the probable source of the sound—the synchronous video. If infants instead spend more time looking at the asynchronous video, this would support the models of infant attention that highlight information-seeking and preferences for moderate complexity levels. Furthermore, we explored how the pitch of the rhythmic sequence might impact infant attention and preference for synchrony. If infants demonstrate low-tone superiority for rhythmic processing, they may detect synchrony more readily in low-frequency conditions.

## 2 Methods

### 2.1 Participants

Full-term infants (>36 weeks gestation) between 8 and 12 months were recruited from the University of Toronto Scarborough Infant and Child Database. Target sample sizes were determined based on laboratory resources and samples used in prior research, documenting infant auditory–visual integration from various research groups (Lewkowicz et al., 2010; Kubicek et al., 2014; Gerson et al., 2015). Data were collected from 44 infants before testing was paused in March 2020 due to COVID-19 laboratory shutdowns. Seven infants were tested but excluded from analysis due to fussiness (4), calibration errors (2), or equipment failure (1). This left data from 37 infants in the analyses (M age = 10.46 months, SD = 1.27; 21 girls, 16 boys). The first 21 participants were assigned to the isochronous rhythm condition. The following 16 participants were assigned to the syncopated rhythm condition.

Infants came from diverse language backgrounds, with 57% exposed to more than one language, and mean English exposure at 77% (1 of the 37 participants did not report language background). Household incomes exceeded medians ($84,000 CAD; Statistics Canada, 2023) reported in this geographic region, with 19% reporting <$60,000/year, 35% reporting between $60,000 and $120,000/year, and 46% reporting > $120,000/year. Two caregivers did not provide income information. Additionally, 46% of caregivers reported that their infants participated in organized music lessons (for example, paid weekly programs such as Kindermusik or Music Together or free community weekly drop-in classes; 5 did not respond).

The University of Toronto Research Ethics Board approved all experimental procedures (Protocol 36642). Informed written consent was obtained from all parents. Infants received a junior scientist t-shirt and certificate for participating.

### 2.2 Stimuli

Auditory stimuli were generated in Audacity (2.2.2) on a Windows computer. These stimuli consisted of 200 ms pure tones with inter-beat intervals (IOI) of 430 ms (100 beats per minute, or bpm) or 600 ms (140 bpm). Pure tones had a 10-ms rise time and a 50-ms fall time. High- and low-frequency patterns were created using pure tone sine waves with 1236.8 Hz and 130 Hz, respectively, consistent with frequencies utilized by Lenc et al. (2018). Isochronous (x-x-x-x-x-x-x-x-) and syncopated (x--x--x---x-x---) rhythm patterns were used.

**Figure 1:**
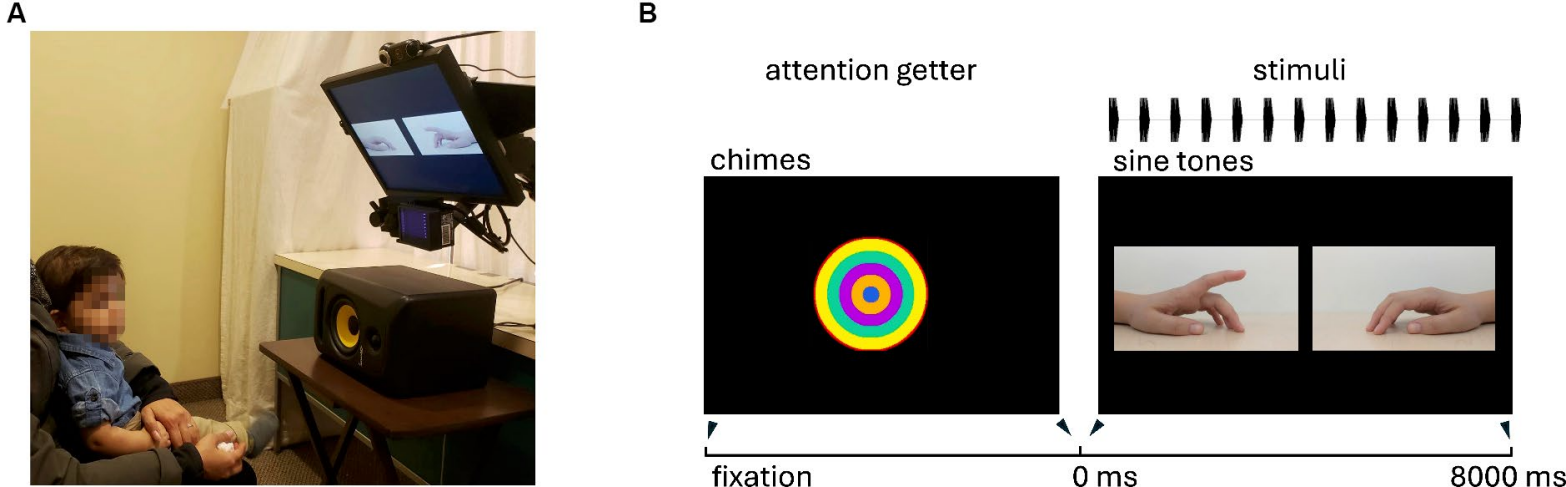
**(A)** Example of experimental setup. Infants sat on their parent’s lap. Calibration stickers were placed on the children’s foreheads. The loudspeaker was directly centered under the display screen and in front of the infant. Note that lights were dimmed during data collection. **(B)** An example of one trial. First, an attention-getter (shifting concentric circles accompanied by a chime sound) is displayed until the infant fixates. Then, the trial begins—two tapping hands are simultaneously presented, angled inward so that the point of contact is equidistant from the prior central fixation. The auditory rhythm is presented via the loudspeaker below the screen. Trials lasted 8000 ms.

In each trial, visual stimuli consisted of two side-by-side finger-tapping videos: one synchronous with the tempo of the auditory stimulus and one asynchronous. Both videos were oriented such that the fingers were pointed toward the middle against a black background (see Figure 1). Pointing the fingers inward ensured that the points of impact were equidistant from the fixation point in the center of the screen, which infants fixated on before the trial began.

These videos were recorded at 60 frames per second using a Google Pixel 2. The model was a white adult woman tapping with her dominant (right) hand and pointer finger. Two types of tapping videos were recorded: isochronous and syncopated, each initially recorded at 515 ms IOI (116.5 beats per minute). These videos were subsequently sped up and slowed down by 16.5% using iMovie (10.1.9) to generate the 430 ms IOI (fast) and 600 ms IOI (slow) versions of each video. Mirror images were created by duplicating and flipping the videos to create a version with the finger pointing to the opposite side. The video commenced with both fingers starting their ascent from the surface (a wooden table) at the same time. Audio files were aligned so that the first pure tone occurred in synchrony with the first impact point for the synchronous video. There were 8 unique stimulus combinations that counterbalanced synchronous video location (left/right), tempo of the auditory rhythm (fast/slow), and pitch of the auditory rhythm (high/low). These 8 trial types were randomized within each trial block. An attention-getter, presented during calibration and between trials, was obtained from the Open Science Framework website,^1^ consisting of colorful concentric circles and auditory chimes.

### 2.3 Apparatus

Infants were tested sitting on their parent’s laps in a small dark room surrounded by heavy white curtains (see Figure 1). Each parent was provided with blacked-out glasses obscuring their vision as well as noise-isolating headphones playing music. Infants sat 55 cm in front of a 1280 × 1024 computer monitor. The audio was presented at 78.8 dBC SPL from a KRK Rokit 5 speaker centered below the monitor. Stimuli were presented using Experiment Builder (SR Research).

Eye movements were recorded using an EyeLink 1000 Plus system (SR Research Ltd.). The eye tracker camera recorded reflections of infrared light on the infant’s cornea in relation to their pupil at a sampling rate of 500 Hz. A head-free setup was utilized with a target sticker placed on the infant’s forehead between their eyebrows. The right eye was tracked across all infants. A three-point calibration procedure with manual experimenter confirmation was used to map gaze position to screen position, using the attention-getter (colorful spinning circles accompanied with a chime) at each target point.

### 2.4 Procedure

Following calibration, the experiment began. The attention-getter was presented in the center of the screen before each trial. The experimenter manually triggered the trial presentation after confirming that the infant gaze was within 10 degrees of the attention-getter and correcting for drift. Following the attention-getter, trials were presented for 8 s. Blocks of the 8 trial types (counterbalanced for synchrony left/right, fast/slow tempo, and high/low pitch) were repeated six times (48 trials total). The trial order was randomized within each block. Once calibration was complete, the procedure took approximately 10 to 15 min.

Upon completion of the experiment, the caregiver completed a general demographics questionnaire and the “Music@Home-Infant” questionnaire (Politimou et al., 2018), which gathered information about infants’ musical home environments.

### 2.5 Data processing

For the analyses below, trials were retained if infants looked at least once at the left and at least once at the right display. These criteria led to the exclusion of 235 out of the 1527 trials (15%). This criterion was selected *a priori* to prioritize trial inclusion. The remaining trials had looking times that ranged from 172 ms to 7903 ms (M = 4432 ms, SD = 1881 ms). Only 11 (<1%) of the included trials had looking times that were less than 2 SDs below the mean (670 ms). To liberally capture infant looking, our interest areas focused on the right vs. left half of the screen rather than specific interest areas in each video.

### 2.6 Analyses

Our primary dependent measures were (1) the proportion of time spent looking at the side of the screen displaying synchronous over the asynchronous display and (2) overall dwell times to either (synchronous/asynchronous) display. Exploratory dependent measures are described in more detail below. The proportion of looking at the synchronous and asynchronous displays was compared to chance levels (0.50) using one-sample t-tests. Linear mixed-effects models (LMEM; glmmTMB package, Brooks et al., 2017) in R (version 4.2.2, R Core Team, 2023) were used to evaluate the effects of pitch, tempo, and rhythmic complexity on infant-looking measures. We contrast-coded the repeated-measures variables pitch (low = −1, high = 1) and tempo (slow = −1, fast = 1) and the between-participants variable rhythmic complexity (isochronous = −1, complex = 1), such that a main effect of a factor represents the average effect across levels of the other factors.

Age, trial, and Music@Home scores were included as continuous predictors. For proportion-looking data, we assumed a beta distribution. For overall looking time, Gaussian distributions were assumed. Random intercepts for participants were included in the models to account for repeated measures.

## 3 Results

### 3.1 Preferential looking to synchronous or asynchronous displays

The infant proportion of time spent looking at the synchronous compared to the asynchronous side of the screen was calculated per trial. Overall, relative to the time infants spent looking at either half of the screen, they spent 49.9% of the time dwelling on the synchronous side. This did not differ significantly from chance levels (50%), *t*(36) = −0.11, *p* = 0.913 (one-sample test). To explore whether this null finding was driven by trials where infant looking may not have been long enough to notice synchrony, we ran the same test using a strict trial inclusion criterion requiring at least 1200 ms (at least two tap cycles) of looking to both the synchronous and asynchronous displays and found the same pattern, *t* (36) = −0.61, *p* = 0.548 (one-sample test). This pattern of distributed attention was consistent across conditions. A linear mixed-effects model demonstrated no significant effects of pitch, tempo, rhythmic complexity, or interaction between these terms on proportion time looking to the synchronous side (*p*’s > 0.467). We also found no significant relationship between Music@Home general factor score, infant age, or trial number and proportion of synchronous looking (*p*’s > 0.479). A simplified model exploring only the interaction between pitch and tempo while accounting for trial number revealed similar findings (*p*’s > 0.282).

### 3.2 Overall attention across trials

The infant’s total looking duration for either display was calculated per trial. A linear mixed-effects model was used to explore whether total looking changed across conditions (pitch, speed, rhythmic complexity, and trial number) and infant characteristics (age and Music@Home scores). While no interactions emerged, we found a simple effect of the trial (*B* = −44.41, *SE* = 3.22, *z* = −13.77, *p* < 0.001) and pitch (*B* = −312.16, *SE* = 140.63, *z* = −2.22, *p* = 0.026). As expected, overall attention to the displays reduced as trials progressed. Interestingly, infants spent more time looking at the screen in the low-pitch audio conditions (*M* = 4435 ms) than in the high-pitch audio conditions (*M* = 4271 ms).

### 3.3 Exploratory analyses around beat windows

Our initial hypothesis—that infants would prefer synchronous or asynchronous displays—was not supported. After completing our planned analyses, we further explored whether infants’ attention to the synchronous and asynchronous hand shifted dynamically around the beat windows. This exploratory analysis was inspired by recent infant eye-tracking work showing that infants selectively attend a singer’s eyes (compared to the mouth) at rhythmically important moments (Lense et al., 2022). For this analysis, 35 ms bins were identified across the window 210 ms before and after each beat for both the fast and slow taps within each trial. Then, for each trial, we determined if each infant fixated on the side of the screen displaying the tapping hand—looking at the fast hand around fast beat windows and the slow hand around slow beat windows—at least once within each of these 35ms bins. We then aggregated looks at the tapping hand around each beat window, considering whether the audio aligned with that beat window. This approach allowed us to calculate the proportion of bins containing looks at the same tapping hand when that hand was either congruent with the audio or incongruent with the audio. From these values, we calculated a difference score reflecting congruent–incongruent looking across bins surrounding the fast and slow beat windows. Positive values reflect more looking to the tapping hand on synchronous compared to asynchronous audio trials. For example, this would mean more looking to the fast hand around the fast beat window when fast audio is presented than when slow audio is presented. If infants did not integrate audiovisual information, they should distribute their attention similarly to a given hand regardless of audio congruence, resulting in a difference score close to 0. However, we hypothesized that if auditory stimuli guide visual attention to the tapping hand, infants should display a greater tendency to look at the tapping hand when it aligns with the audio.

**Figure 2:**
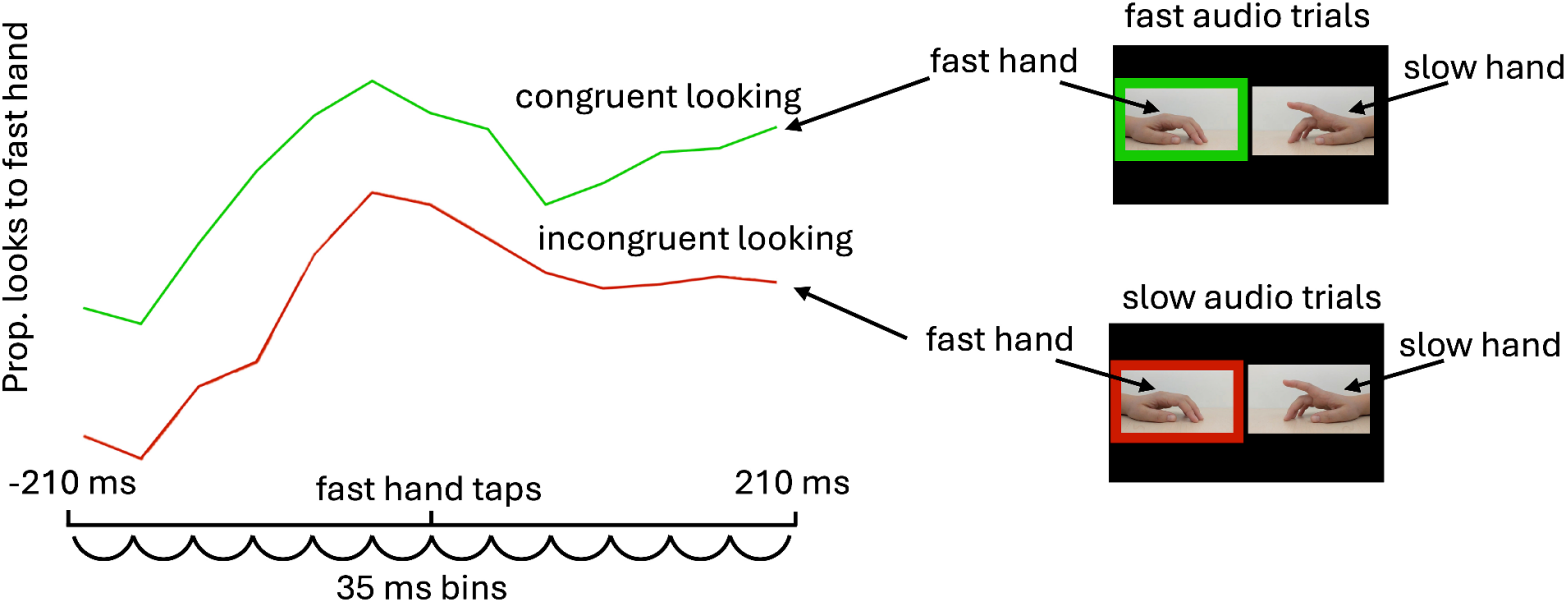
The exploratory analysis investigated whether attention to the stimuli was enhanced by audiovisual congruence around the tap window. Specifically, we asked whether attention around taps for a given video (e.g., the fast hand) was enhanced when the audio was congruent compared to incongruent. First, the window around each finger tap was divided into 35-ms bins (6 before and 6 after). Each bin was assigned 1 or 0 (1 = a fixation to the tapping hand occurred). These values were then aggregated across taps within trials and across trials within each pitch condition. Finally, the proportion of bins containing looks at the tapping hand on congruent trials (in this example, the fast hand in fast audio trials) was calculated and compared to the proportion of bins containing looks at the tapping hand on incongruent trials (here, the fast hand in slow audio trials). The difference scores in Figure 3 reflect incongruent looking subtracted from congruent looking.

A linear mixed-effects model was used to explore whether an infant looking at the congruent tapping hand was guided by features of the auditory stimuli. Our model explored the simple effects and interactions between pitch (high and low), speed of the tapping hand (fast and slow), and rhythmic complexity (isochronous and complex). A three-way interaction emerged, *B* = −0.07, *SE* = 0.01, *z* = −4.89, *p* < 0.001. Simple effects were explored within each rhythmic complexity condition (see Figure 2).

**Figure 3:**
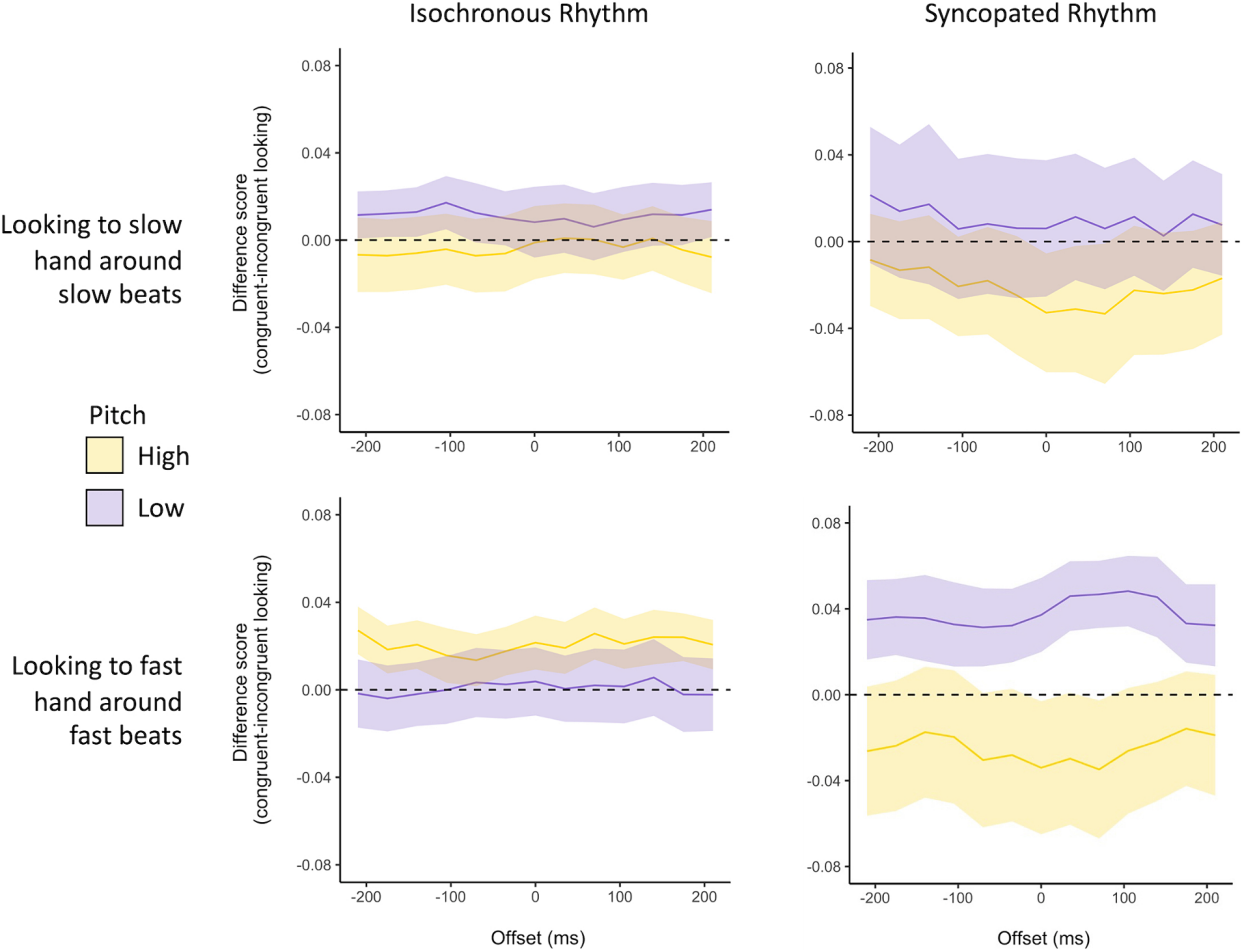
Here, we plot infant attention to the tapping fingers around the fast (top row) and slow (bottom row) beat windows for infants in the isochronous (left) and complex (right) rhythm conditions. The y-axis shows the difference score in looking at these hands when the audio is congruent vs. incongruent with that hand’s tapping tempo (i.e., looking above baseline represents more looking when audio aligns than when audio does not align). The error bars represent the standard error of the mean.

Within the isochronous rhythm condition, the main effects of pitch (*B* = −0.02, *SE* = 0.005, *z* = −3.12, *p* = 0.002) and speed of the tapping hand (*B* = −0.01, *SE* = 0.005, *z* = −2.18, *p* = 0.029) were qualified by an interaction between these factors (*B* = 0.04, *SE* = 0.007, *z* = 5.09, *p* < 0.001). Above-baseline congruent looks (more looking when audio is congruent) to the fast hand were greater in the high-pitch condition than in the low-pitch condition, *p* < 0.001. Conversely, above-baseline congruent looks at the slow hand were greater in the low-pitch condition than in the high-pitch condition, *p* <. 001.

In the syncopated rhythm condition, the main effects of pitch (*B* = −0.03, *SE* = 0.009, *z* = −3.50, *p* < 0.001) and speed of the tapping hand (*B* = 0.03, *SE* = 0.009, *z* = 3.09, *p* = 0.002) were again qualified by an interaction between these factors (*B* = −0.03, *SE* = 0.01, *z* = −2.47, *p* = 0.014). The difference scores for looks at the fast-tapping hand and the slow-tapping hand were both greater in the low-pitched conditions than in the high-pitched conditions. However, this pitch effect was more dramatic for congruent looking at the fast hand. Visual inspection suggests that variability across participants was higher in this condition than in the isochronous rhythm condition. While this increased variability may be reflective of the increased complexity of the stimulus, it may also be a by-product of the smaller sample (n = 16 compared to 21 infants).

## 4 Discussion

When presented with two side-by-side videos of fingers tapping rhythmically, 8- to 12-month-old infants did not show overall within-trial preferences for the video that aligned with the auditory rhythm. Furthermore, our analyses found no effect of rhythmic complexity (isochronous/syncopated), auditory pitch (high/low), tempo (fast/slow), or infant musical background on their interest in the synchronous vs. asynchronous display. This finding may be surprising, given that much younger infants prefer to attend to visual displays that align with presented audio (Spelke, 1979; Lewkowicz et al., 2010), but converges with other research within this age group, suggesting that this synchrony preference is inconsistent, if present at all (Kubicek et al., 2014; Lewkowicz et al., 2015). These findings are unlikely to reflect low interest in the stimuli, which are arguably less interesting than speech streams—trial-level dwell times exceeded 50% of the trial lengths.

A synchrony preference would have provided support for the auditory scene analysis framework (Bregman, 1994) and would have suggested that infants use auditory–visual synchrony to guide attention to likely sound sources. Overall preferences for attending asynchronous displays, on the other hand, would have suggested that infants in this age range find synchrony detection to be trivial and shift to the display, warranting more exploration (Hunter and Ames, 1988).

Not finding support for either model and inspired by recent research investigating infant attention to a singing face (Lense et al., 2022), we explored infant attention around the beat window. Specifically, we asked if cross-trial interest in the fast and slow displays was facilitated by hearing a congruent rhythm. Here, our analysis revealed preliminary evidence for integration and evidence that stimulus features mattered. Across most conditions, infants displayed a greater tendency for congruent compared to incongruent fixations to the tapping hand in the low-pitch condition compared to the high-pitch condition. This pattern was particularly pronounced in the syncopated rhythm/fast hand condition. These initial findings provide preliminary evidence that infants are integrating the rhythms that they hear with the rhythms that they see—if these streams were being processed independently, we would not expect to see above-baseline congruent looks. Above-baseline looking suggests that infants are especially likely to look at a particular rhythmic visual display when it aligns with the rhythms being heard. While this analysis is exploratory, it highlights the value of exploring fine-grained infant attention to synchronous displays instead of only looking at averaged interest collapsed across trial lengths.

We did not find any evidence for individual differences in synchrony preferences or congruent looks around beat windows relating to infant age or home music background (from the Music@Home scale). Due to the interruption of in-person data collection by the COVID-19 lockdowns, future research with larger samples may be able to address this question more directly. For example, previous research with 6-month-old infants shows that infants provided an opportunity to interact with a toy drum are subsequently more interested in videos showing the same toy drum being struck synchronously rather than asynchronously with auditory rhythms (Gerson et al., 2015). This example of short-term experience raises questions about whether long-term experience also impacts early attentional biases for audiovisual synchrony.

Irrespective of whether infants engaged in synchronous or asynchronous looking, they demonstrated more time attending to the visual displays in the low-pitch condition compared to the high-pitch condition. This observation may be interpreted in light of the low-tone superiority effect, demonstrating that rhythmic information is better extracted from low-pitch signals (Hove et al., 2014; Lenc et al., 2023). Perhaps infants were more interested in exploring the two visual rhythms when the auditory stream provided a more salient rhythmic context. Future research could explore the effect of pitch on rhythm processing by asking whether infants are better able to detect rhythmic violations in low- compared to high-pitch streams. It is also worth noting that infant preferences for pitch in musical signals are context-dependent—for example, infants prefer to listen to low- over high-pitched lullabies but prefer to listen to high- over low-pitched playsongs (Volkova et al., 2006; Tsang and Conrad, 2010). Lullabies also tend to have slower and steadier rhythms (Trainor et al., 1997) and are more effective at downregulating infant arousal (Cirelli et al., 2020). Questions remain about how pitch interacts with rhythm and functional goals in shaping infants’ perceptions and emotional reactions to everyday musical exchanges.

Future studies are needed to harmonize the existing research on the developmental trajectory of auditory–visual integration in infancy. Here, we opted to utilize musically relevant rhythmic patterns (isochronous and syncopated), which were selected to match those used in prior work exploring the low-tone superiority effect (Lenc et al., 2018). One potential consideration, however, is that the audiovisual pairings we selected—namely, sine tones and tapping fingers—do not occur naturally. Previous research has shown that infants as young as 6 months are sensitive to some aspects of audiovisual congruence in impact events. When presented with side-by-side videos that are *both* temporally synchronized with an auditory stimulus, infants preferentially watch the display that matches the acoustic properties of the heard material (Bahrick, 1987). In contrast, however, infants are likely to integrate natural speech and sine wave speech when presented synchronously with a talking face (Baart et al., 2014) and experience audiovisual illusions—such as the sound-bounce illusion—even when the “sound” paired with the bounce is an artificial beep (Sekuler et al., 1997; Scheier et al., 2003). Therefore, many unanswered questions remain about the potential facilitatory effects of naturalistic vs. artificial audiovisual pairing and the role of experience in informing infants’ expectations about naturalistic audiovisual pairings. The present research highlights that considering stimulus properties and tracking dynamic attention is an important step toward building predictions about how audiovisual synchrony guides attention in early life.

## Data availability statement

The raw data supporting the conclusions of this article will be made available by the authors, without undue reservation.

## Ethics statement

The studies involving humans were approved by the University of Toronto Research Ethics Board. The studies were conducted in accordance with the local legislation and institutional requirements. Written informed consent for participation in this study was provided by the participants’ legal guardians/next of kin.

## Author contributions

LC: Conceptualization, Data curation, Formal analysis, Funding acquisition, Investigation, Methodology, Resources, Supervision, Visualization, Writing – original draft, Writing – review & editing. LT: Conceptualization, Investigation, Methodology, Writing – review & editing. HK: Conceptualization, Data curation, Formal analysis, Investigation, Methodology, Supervision, Visualization, Writing – review & editing.

## Footnotes

^1^ https://osf.io/wh7md/
